# A Three-Gene Signature for Predicting the Prognosis of Patients Treated with Transarterial Chemoembolization (TACE) and Identification of PD-184352 as a Potential Drug to Reverse Nonresponse to TACE

**DOI:** 10.1155/2022/2704862

**Published:** 2022-09-28

**Authors:** Zicong Xia, Wenjing Zhao, Jibin Liu, Jing Zhang, Jing Pan, Kang Chen, Lele Wang, Hui Zhao, Xiaoqing Chen

**Affiliations:** ^1^Department of Interventional Radiology, Affiliated Hospital of Nantong University, Medical School of Nantong University, Nantong, 226001 Jiangsu, China; ^2^Cancer Research Center Nantong, Tumor Hospital Affiliated to Nantong University, Nantong, 226361 Jiangsu, China; ^3^Research Center of Clinical Medicine, Talents Office of Party Committee and Human Resources Department, Affiliated Hospital of Nantong University, Nantong, 226001 Jiangsu, China

## Abstract

**Background:**

Transarterial chemoembolization (TACE) is a first-line treatment for patients with unresectable hepatocellular carcinoma (HCC). Owing to differences in its efficacy across individuals, determining the indicators of patient response to TACE and finding approaches to reversing nonresponse thereto are necessary.

**Methods:**

Transcriptome data were obtained from the GSE104580 dataset, in which patients were marked as having TACE response or nonresponse. We identified differentially expressed genes (DEGs) and performed Kyoto Encyclopedia of Genes and Genomes (KEGG) analysis. We screened genes with a prognostic value for TACE in the HIF-1 signaling pathway by univariate regression analysis. By using least absolute shrinkage and selection operator (LASSO) Cox regression, we established a multigene signature in GSE14520, which we verified using a drug sensitivity test. The Connectivity Map (CMap) database was used to find potential drugs to reverse nonresponse to TACE.

**Results:**

We constructed a prognostic signature consisting of three genes (erythropoietin (*EPO*), heme oxygenase 1 (*HMOX1*), and serine protease inhibitor 1 (*SERPINE1*)) that we validated by drug sensitivity test. After dividing patients treated with TACE into high- and low-risk groups based on this new signature, we showed that overall survival (OS) of the high-risk group was significantly lower than that of the low-risk group and that the risk score was an independent predictor of OS in patients treated with TACE. Based on our CMap findings, we speculated that PD-184352, an inhibitor of mitogen-activated protein kinase (MEK), had potential as a drug treatment to reverse nonresponse to TACE. We confirmed this speculation by using PD-184352 in a cell promotion experiment in a TACE environment.

**Conclusion:**

We constructed a TACE-specific three-gene signature that could be used to predict HCC patients' responses to and prognosis after TACE treatment. PD-184352 might have potential as a drug to improve TACE efficacy.

## 1. Introduction

Hepatocellular carcinoma (HCC) is one of the most common malignant tumors, with the fifth highest incidence rate and the third highest mortality rate [1]. More than two thirds of HCC patients cannot undergo surgical resection [2]. According to the Barcelona clinic liver cancer (BCLC) staging system, transarterial chemoembolization (TACE) is recommended as first-line treatment for midstage or advanced HCC [3, 4]. Although TACE can improve outcomes in some patients, the objective remission rate after the first TACE treatment is less than 40% [5]. Therefore, it is particularly important to reveal the causes of TACE nonresponse and distinguish patients who cannot benefit from TACE before surgery.

Professor Wang [6] of the American Cancer Research Center believes that hypoxia is a potential drug resistance mechanism of TACE nonresponse. In TACE nonresponse tumors, hypoxia reprogramming may have been experienced before TACE treatment or it may lead to enhanced response to hypoxia caused by TACE. The HIF (hypoxia-inducible factor) family is significantly upregulated in a hypoxic environment, abnormal activation of the HIF-1 signaling pathway plays an important role in tumor progression, HIF-dependent gene expression controls epithelial mesenchymal transformation (EMT), invasion, migration, and angiogenesis [7]. TACE causes the hypoxic microenvironment of the tumor, and researchers have shown that the expression of HIF-1*α* is significantly increased after TACE and is related to the poor prognosis of TACE [8, 9], so the current research on TACE nonresponse mainly focuses on the abnormal activation of the HIF-1 signaling pathway. Our study also found that the HIF-1 signaling pathway is enriched in TACE nonresponse, which is why we decided to further study the HIF-1 signaling pathway.

Due to the heterogeneity of HCC, patient response to TACE is variable. Therefore, there is continuous interest in preoperative screening of those who could benefit from this procedure [10]. In recent years, scoring systems have been designed based on certain routine examination items to predict prognosis in TACE, such as the hepatoma arterial embolization (HAP) prognostic score, which applies simple laboratory and imaging indicators [11]. The HAP score has the advantages of ease of application and simplicity; however, because its calculation is based only on certain macroscopic data, it cannot reflect deeper indicators of prognosis after TACE treatment, which is a disadvantage in the era of precision medicine. Furthermore, the most current scoring systems are limited by their lack of specificity to TACE. Therefore, developing a method to assess whether patients would benefit from TACE before they are ready for such treatment could improve survival in certain patients.

It is important to clarify why some patients do not benefit significantly from TACE. In this study, using a Gene Expression Omnibus (GEO) dataset, we established a signature consisting of three genes (*EPO*, *HMOX1*, and *SERPINE1*) to predict the prognosis in TACE. We divided cell lines into TACE response and nonresponse groups according to the gene signature and then evaluated the differences between the two groups to verify the signature's effectiveness. Using the Connectivity Map (CMap) database, we screened PD-184352, an inhibitor of mitogen-activated protein kinase (MEK), and proved that it could improve the sensitivity of HCC cells and the effects of TACE treatment *in vitro*. We believe that our initial exploration of a new intraoperative or postoperative administration pattern for TACE is worthy of further clinical trials.

## 2. Methods

### 2.1. Patient Cohorts and Cell Lines

We screened genes in the HIF-1 signaling pathway from the GSE104580 cohort. This dataset contains data from 147 patients treated with TACE for whom TACE was the primary treatment. Of these 147 patients, 81 responded well (TACE response) and 66 responded poorly (TACE nonresponse) [12]. Ribonucleic acid (RNA) extracted from tumor biopsies of HCC patients to be treated with TACE was analyzed using Affymetrix gene arrays (Affymetrix, Santa Clara, CA, USA).

From the GSE14520 development cohort, we selected 74 patients with liver cancer who had received adjuvant TACE after liver resection and 30 who had received TACE after recurrence. In addition, we included 85 patients who had undergone liver resection alone. The details of this cohort were reported by Fako et al. [6].

Transcriptomic data of liver cancer cell lines were obtained from the Cancer Cell Line Encyclopedia (CCLE). We divided cell lines into responder-like and nonresponder-like groups by the calculating risk score based on the transcriptomic data described above.

### 2.2. Establishment of a Potential Prognostic HIF-1 Signaling Pathway-Related Gene Signature

Gene expression data were normalized by formula log_2_ using the limma package [13] in R software v4.1.0 (R Foundation for Statistical Computing, Vienna, Austria) to calculate differentially expressed genes (DEGs) between the TACE response and nonresponse groups in GSE104580 under the following conditions: Benjamini–Hochberg adjust *P* < 0.05, |log_2_fold change (FC)| > 0.5, and *P* < 0.05. We then performed Kyoto Encyclopedia of Genes and Genomes (KEGG) analysis of the dysregulated genes in the TACE nonresponse group using the clusterprofiler package in R [14]. Seventeen genes enriched in the HIF-1 signaling pathway were selected and then analyzed by univariate regression in GSE14520. Finally, we screened eight survival-related genes: aldolase, fructose-bisphosphate C (*ALDOC*); egl-9 family hypoxia-inducible factor 3 (*EGLN3*); enolase 2 (*ENO2*); *EPO*; hexokinase 2 (*HK2*); *HMOX1*; 6-phosphofructo-2-kinase/fructose-2,6-biphosphatase (*PFKFB3*); and *SERPINE1*. Overall survival (OS) and recurrence-free survival (RFS) were calculated using the Kaplan–Meier (K–M) method; the survival package was used for statistical analysis and survminer package was used for visualization. With the glmnet package, we performed least absolute shrinkage and selection operator (LASSO) regression and established a prognostic signature to compress some regression coefficients, preserving the benefits of subset shrinkage [15, 16]. Subsequently, we established the following formula to calculate the risk score:

(1) $$\begin{array}{c} \text{Risk score}=\overset{n}{\underset{i=1}{\sum }}\left[\text{expression of gen}{\text{e}}_{i}\times \beta i\right], \end{array}$$

where *n* represents the number of genes in the signature and *β* represents the coefficient of genes obtained from LASSO regression.

### 2.3. Establishment of a Protein–Protein Interaction Network

We explored possible interactions between proteins with data from the Search Tool for the Retrieval of Interacting Genes/Proteins (STRING). Then, we established a protein–protein interaction network (PPI) by visualizing that data using Cytoscape v3.9.1 (https://cytoscape.org/).

### 2.4. Cell Lines

We added 10% fetal bovine serum (FBS) and 1% penicillin–streptomycin to Dulbecco's modified Eagle medium (DMEM) (Suzhou Meilunbio, Suzhou, China) to culture Hep G2 and Hep 3B cells.

### 2.5. Detection of Potential Compounds

The CMap database (https://clue.io/CMap) matches responses in cell line transcriptome data to different drugs to screen for the potential use of such drugs in medical treatments [17]. We input significantly upregulated genes of the high-risk group in GSE14520 into the L1000 platform of CMap to find compounds that might cause reversal reactions. Subsequently, we took compounds with connectivity scores of less than −98, which could cause the opposite reaction of the Hep G2 cell line, as drugs that could potentially reverse nonresponse to TACE.

### 2.6. Quantitative Real-Time PCR (RT-PCR)

We use RNA Isolation Kit V2 (Vazyme, RC112-01, Nanjing, China) to extract the RNA of Hep G2 and Hep 3B, which was then synthesized complementary DNA (cDNA) by using the cDNA Synthesis Kit (Vazyme, R323-01, Nanjing, China). The AceQ Universal SYBR qPCR Master Mix (Vazyme, Q511-02/03, Nanjing, China) was utilized to perform RT-PCR on the SteponePlus (Applied Biosystems, Thermo Fisher, Waltham, MA, USA). Relative expression values were normalized to the control gene *β*-actin. The primer pairs used in this study are shown in Table 1. The relative expression of these genes was calculated by the 2^−ΔΔ*CT*^ method.

### 2.7. Cell Viability and Drug Sensitivity

We added 5000 cells/well to 96-well plates and diluted them with DMEM. After 24 h of continuous culture in a 5% CO_2_ incubator at 37°C, we injected different doses of lobaplatin (0, 2, 4, 8, 16, 32, and 64 *μ*g/mL) or PD-184352 (0, 0.1, 0.2, 0.4, 0.8, 1.6, and 3.2 *μ*M) into each well under both normoxic and hypoxic conditions for 48 h. Then, we added 10 *μ*L water-soluble tetrazolium 8 (WST-8; #A311; Beyotime Institute of Biotechnology, Shanghai, China) to each well and cultured the cells for another 2 h. An enzyme-linked immunosorbent assay (ELISA; Multiskan FC; Thermo Fisher, Waltham, MA, USA) was used to measure the optical density (OD) of each sample at 450 nm. We used the DRC package in R [18] to analyze OD values in order to calculate half-maximal inhibitory concentration (IC_50_) and the ggplot2 package [19] for visualization.

### 2.8. Cell Proliferation Assays

We added 5000 cells/well to 96-well plates and diluted them with DMEM; each group had eight duplicate wells. After 24 h of continuous culture in a 5% CO_2_ incubator at 37°C, cells were treated with IC_50_ lobaplatin, IC_50_ PD-184352, or both under hypoxic conditions for different time periods (0, 12, 24, 36, and 48 h). Every 12 h, we added 10 *μ*L WST-8 to each well and cultured the cells for another 2 h. Multiskan FC was used to measure the OD value of each sample at 450 nm, and OD values were plotted in GraphPad Prism v8.0.2 (GraphPad Software Inc., San Diego, CA, USA).

### 2.9. Statistical Analysis

All statistical tests were two tailed, and we considered *P* < 0.05 statistically significant. Univariate and multivariate Cox regression models were applied to confirm characteristics that were valuable in predicting the prognosis of patients treated with TACE. We used an independent *t*-test to assess differences between the two groups. A correlation matrix was constructed with ggplot2. To calculate statistics for the experimental data, we used GraphPad Prism 8.0.2.

## 3. Results

### 3.1. The HIF-1 Signaling Pathway Was Enriched in the TACE Nonresponse Group

**Figure 1:**
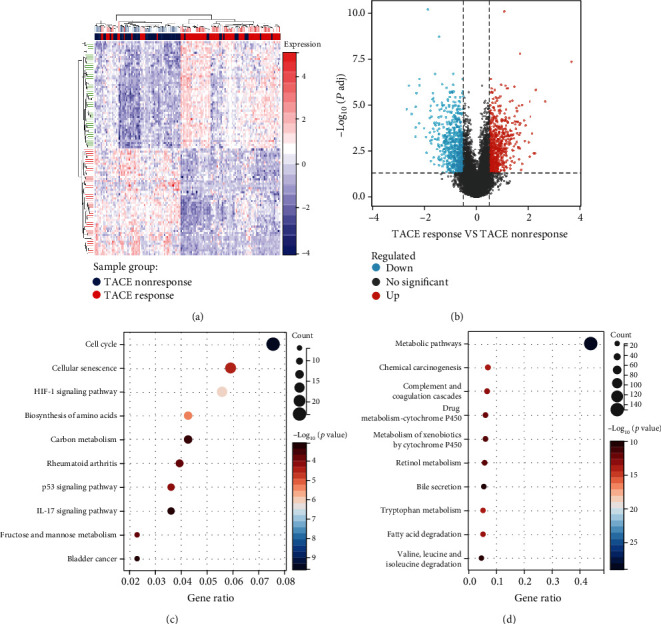
Differences in transcriptome samples between the TACE response and nonresponse groups in GSE104580. (a) The heat map shows the clustering of TACE responders and nonresponders. (b) The volcano map shows genes upregulated in both groups. The bubble diagram shows KEGG pathways enriched in the TACE (c) nonresponse and (d) response groups.

By analyzing differences in gene expression between 81 TACE nonresponders and 66 TACE responders in GSE104580, we identified 735 genes that were upregulated in nonresponders and 749 that were upregulated in responders (|log_2_FC| > 0.5, *P* < 0.05). Based on these genes, we drew a heat induction map (Figure 1(a)) and a volcano map (Figure 1(b)). Then, we performed KEGG enrichment analysis on all 1484 genes and found that the HIF-1 signaling pathway was significantly enriched in TACE nonresponders (Figure 1(c)) but not in TACE responders (Figure 1(d)). Since cutting off the blood supply is an important way for TACE to kill cancer cells, the hypoxia microenvironment is one of the most important features after TACE. The hypoxia microenvironment is an important factor in the activation of the HIF-1 signaling pathway. More importantly, many studies have proved that HIF-1*α* is closely related to poor prognosis in TACE, so we chose the HIF-1 signaling pathway as our next research focus.

### 3.2. Identification of HIF-1 Signaling Pathway-Related Genes Associated with Prognosis of TACE in GSE14520

**Figure 2:**
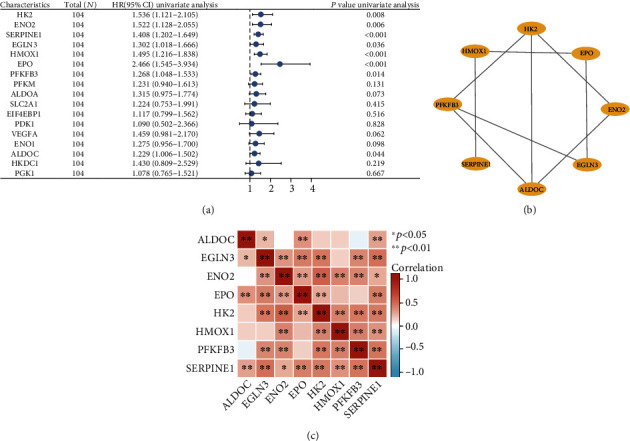
Eight genes related to the probability of overall survival (OS) in GSE14520 and their interactions and correlation relationships. (a) The forest map in the table shows that 8 of the 17 genes were related to poor OS in patients treated with TACE. HR: hazard ratio; CI: confidence interval. (b) A PPI of these eight molecules was plotted using Cytoscape. (c) The correlation heat map of these genes in the TACE nonresponse group of GSE104580.

A total of 17 genes were enriched in the HIF-1 signaling pathway: *ALDOA*, *ALDOC*, EIF4EBP1, *ENO1*, *ENO2*, *EPO*, *HK2*, *HMOX1*, *SERPINE1*, PDK1, *PFKFB3*, *PFKM*, *PGK1*, *SLC2A1*, *VEGFA*, *HKDC1*, and *EGLN3*. Subsequently, using univariate regression analysis in GSE14520, we found that eight genes were associated with poor prognosis of patients treated with TACE (Figure 2(a)).  Figure 2(b) shows the PPI of these eight molecules. We obtained their correlation matrix by performing a correlation analysis on the GSE14520 dataset (Figure 2(c)).

### 3.3. Construction of the Prognostic Signature

**Figure 3:**
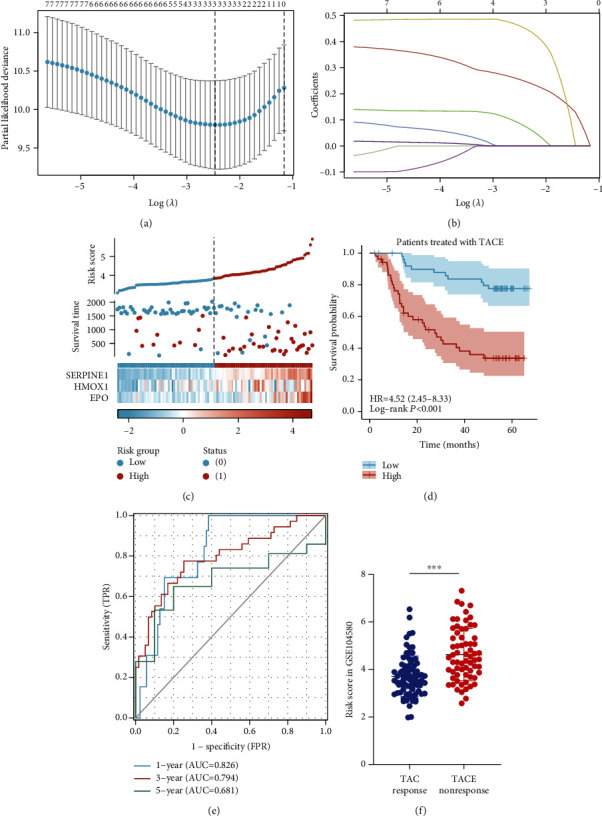
Construction of the prognostic signature. (a) A coefficient profile plot was generated against the log (*λ*) sequence. (b) LASSO coefficient profiles of the eight genes associated with poor prognosis of patients treated with TACE. The coefficient of three genes was not 0 in GSE14520. (c) Risk scores of patients treated with TACE in GSE14520 and median risk score. (d) The K–M curve shows that OS was lower in the high-risk than in the low-risk group in patients treated with TACE in GSE14520. (e) The area-under-the-curve (AUC) value of the time-dependent receiver operating characteristic (ROC) curve shows the prognostic performance of the signature in GSE14520. (f) Difference in risk score between TACE response and nonresponse groups in GSE104580.

Next, we performed LASSO regression analysis to screen the appropriate genes from the abovementioned eight in order to determine their prognostic characteristics (Figures 3(a) and 3(b)). The following formula was used to calculate the risk score:

(2) $$\begin{array}{c} \text{Risk score}=\text{SERPINE}1\times 0.252860249076791+\text{HMOX}1\times 0.0830953460565714+\text{EPO}\times 0.458080063833719. \end{array}$$

We divided patients into high-risk (*n* = 52) and low-risk (*n* = 52) groups based on the median risk score (Figure 3(c)). K–M curve analysis showed that the high-risk group had a poorer prognosis than the low-risk group (Figure 3(d); *P* < 0.001). Time-dependent receiver operating characteristic (ROC) curves showed the ability of our signature to predict prognosis in TACE. The area under the curve (AUC) reached 0.826, 0.794, and 0.681 at 1, 3, and 5 years, respectively, indicating that the prognostic signature showed excellent predictive ability (Figure 3(e)). Then, we used the same formula to calculate the risk scores of samples in the GSE104580 dataset in order to detect any differences between TACE nonresponders and responders. As shown in Figure 3(f), the risk score in the TACE nonresponse group was higher than that in the TACE response group (*P* < 0.0001; 95% confidence interval (CI), 0.5971–1.235).

### 3.4. Our Signature Was Specific to TACE

**Figure 4:**
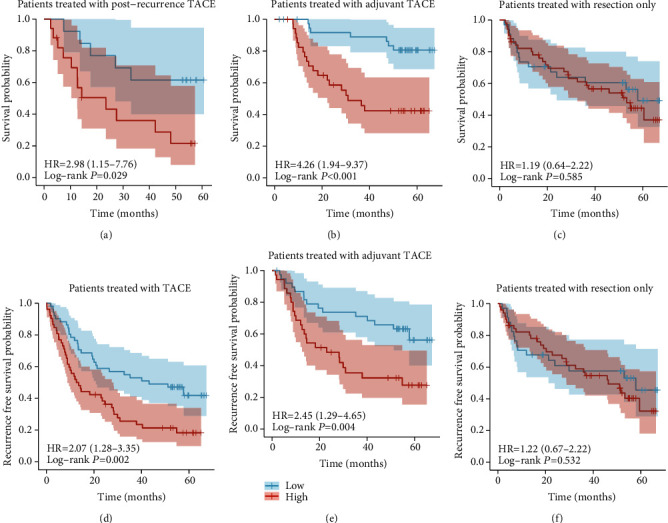
Survival analysis of patients with different treatment methods. OS of patients treated with (a) postrecurrence TACE, (b) adjuvant TACE, and (c) resection only. Recurrence-free survival (RFS) of patients treated with (d) postrecurrence or adjuvant TACE, (e) adjuvant TACE, and (f) resection only.

To verify whether this signature was specific to patients treated with TACE rather than applicable to all HCC patients, we divided patients treated with postrecurrence TACE, adjuvant TACE, and resection only into high- and low-risk groups by median risk score in GSE14520. One hundred four patients were treated with TACE, 74 with adjuvant TACE, and 30 with postrecurrence TACE. K–M curve analysis showed that the high-risk group had a poorer OS than the low-risk group among patients treated with postrecurrence TACE (Figure 4(a)) and adjuvant TACE (Figure 4(b)). However, we saw no difference in patients treated with resection only (Figure 4(c)). Then, we analyzed recurrence-free survival (RFS), except for patients treated with postrecurrence TACE, because the recurrence time is measured from resection to recurrence. As expected, the high-risk group had poorer RFS than the low-risk group among patients treated with TACE (Figure 4(d)) and adjuvant TACE (Figure 4(e)). There was still no difference in patients treated with resection only (Figure 4(f)). These results showed that our signature was specific to TACE.

### 3.5. Verification of the Signature's Distinguishing Ability in Different Clinical Subgroups

**Figure 5:**
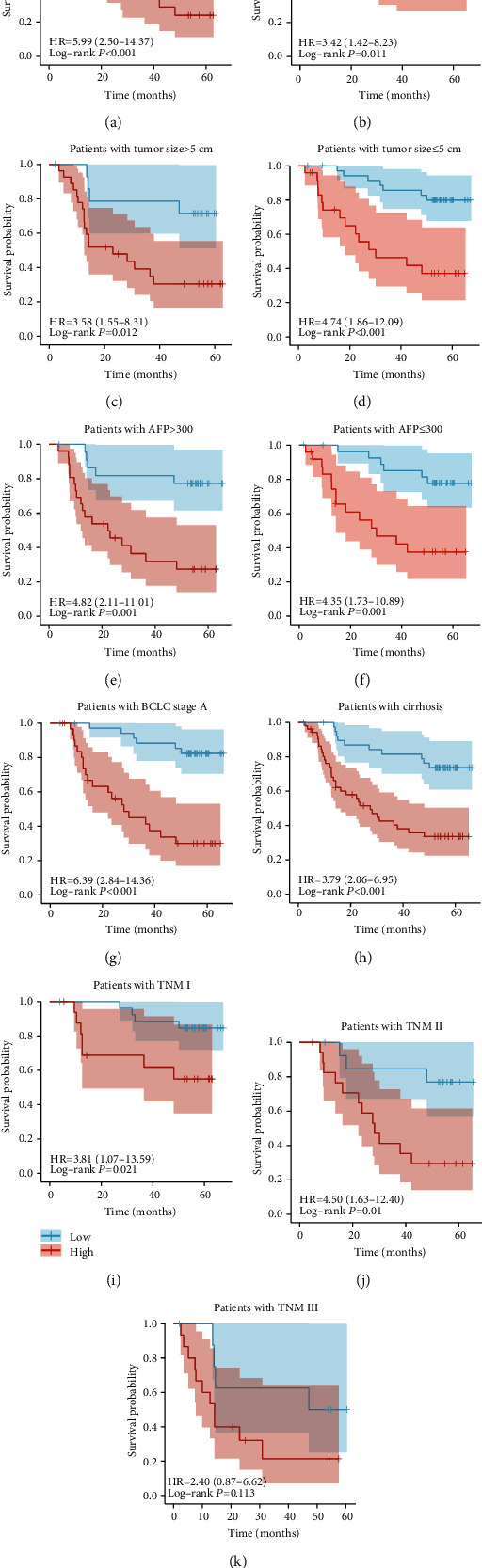
Verification of the signature's distinguishing ability in different clinical subgroups of GSE14520. (a–i) K–M curves for differences in OS among different clinical subgroups of patients in GSE14520 treated with TACE (age ≤ 50 and >50; tumor size > 5 cm; AFP > 300 ng/mL; Barcelona Clinic Liver Cancer (BCLC) staging system stage A; cirrhosis; TNM stages I, II, and III).

Next, we verified the signature's distinguishing ability in different clinical subgroups. To this end, we divided patients treated with TACE into subgroups according to their clinical characteristics (age ≤ 50 and >50; tumor size ≤ 5 and >5 cm; AFP > 300 ng/mL; BCLC stage A; cirrhosis; TNM stages I, II, and III). K–M curves for OS after such division showed that significant differences remained in all subgroups except for TNM III, suggesting that this signature also had a good ability to distinguish between different clinical subgroups (Figure 5(a)–5(k)). Because we enrolled too few patients with no cirrhosis, BCLC stage B, or BCLC stage C, we did not analyze OS for these patients.

### 3.6. Independent Prognostic Value of the Signature

**Figure 6:**
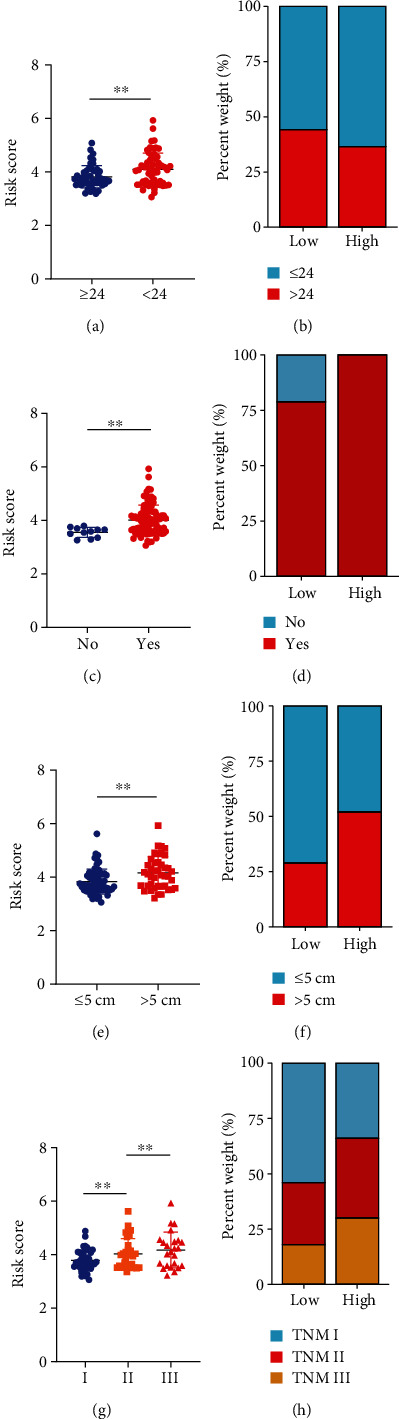
Differences in risk score among different clinical subgroups of patients treated with TACE. With reductions in recurrence months (a, b), cirrhosis (c, d), tumor size (e, f), and TNM staging (g, h), the risk score showed an upward trend.

To illustrate the clinical application value of this signature, we compared the risk scores of various clinical subgroups in GSE14520. That of patients with recurrence within 24 months was higher than that of patients with recurrence after 24 months (Figures 6(a) and 6(b)), which was consistent with our clinical observations. The risk score of patients with cirrhosis of the liver was higher than that of patients without (Figures 6(c) and 6(d)), and patients with tumor size > 5 cm had a higher risk score than those with tumor size ≤ 5 cm (Figures 6(e) and 6(f)), which suggested that TACE offered better prognosis in patients with tumor size ≤ 5 cm and those without cirrhotic livers. In addition, we found remarkable differences among different TNM grades: risk score increased along with the TNM grade, indicating that the signature could effectively distinguish different TNM grades of HCC and therefore might be a potential biomarker of this cancer (Figures 6(g) and 6(h)).

We performed univariate Cox analyses to explore the predictive value of a series of clinical metrics and risk score in GSE14520. As shown in Table 2, a larger main-tumor size (>5 cm), higher TNM stage (III), higher BCLC stage (stages B and C), recurrence months (≤ 24), and risk score are significant risk factors. We then performed multivariate Cox analysis including these four variables and found that the risk score remained an independent predictor (hazard ratio (HR) = 2.109; 95% CI, 1.287–3.455; *P* = 0.003). Recurrence time was also an independent prognostic factor (HR = 0.113; 95% CI, 0.046–0.276; *P* < 0.001).

### 3.7. Verification of the Signature's Predictive Efficacy in Cell Lines

**Figure 7:**
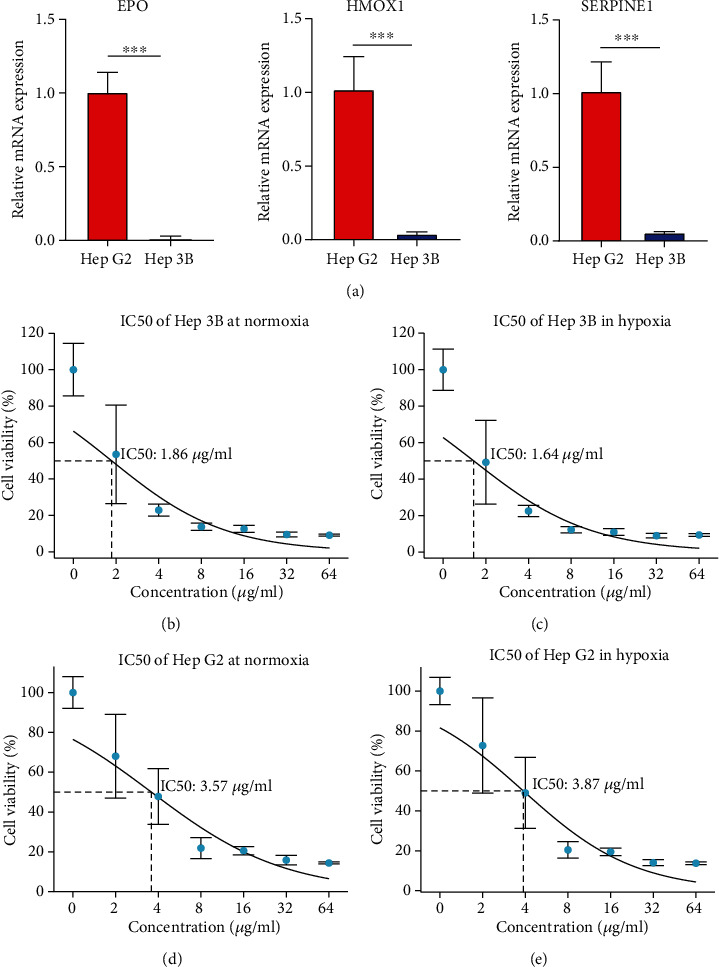
Efficacy of the prognostic signature as verified by drug sensitivity test. (a) The relative mRNA expression of *EPO*, *HMOX1*, and *SERPINE1* in Hep G2 and Hep 3B. (b, c) IC_50_ of lobaplatin on Hep 3B was detected in normoxic and hypoxic environments. (d, e) IC_50_ of lobaplatin on Hep 3B was detected in normoxic and hypoxic environments.

To investigate resistance to TACE, we downloaded the expression data of *EPO*, *HMOX1*, and *SERPINE1* in 25 hepatoma cell lines from the CCLE database and used our model to calculate the risk score of each cell line based on this transcriptomic data. Taking the median of the risk score (Figure 3(c)) as the threshold, we divided each cell line into TACE response and TACE nonresponse and we treated Hep 3B as a responder-like cell line and Hep G2 as a nonresponder-like cell line. The RT-PCR results showed that the relative mRNA expression of EPO, HMOX1, and SERPINE1 in Hep G2 is much higher than that in Hep 3B. To verify the effectiveness of the signature, we tested the IC_50_ of lobaplatin, a platinum chemotherapeutic drug commonly used in TACE, under both normoxia and hypoxia to mimic an environment caused by TACE. We showed that the IC_50_ of lobaplatin in Hep 3B was 1.861 (range, 1.153–2.57) *μ*g/mL under normoxia (Figure 7(a)) and 1.643 (range, 1.017–2.268) *μ*g/mL under hypoxia (Figure 7(b)), while the IC_50_ of lobaplatin in Hep G2 was 3.575 (range, 2.718–4.431) *μ*g/mL under normoxia (Figure 7(c)) and 3.87 (range, 2.999–4.741) *μ*g/mL under hypoxia (Figure 7(d)). The nonresponder-like cell line Hep G2 had a higher IC_50_ than the responder-like cell line Hep 3B under both conditions, and the IC_50_ of lobaplatin on HepG2 increased slightly under hypoxia. Taken together, these results showed that our risk score could distinguish between TACE responders and nonresponders.

### 3.8. PD-184352 Could Be Used to Reverse Nonresponse to TACE

**Figure 8:**
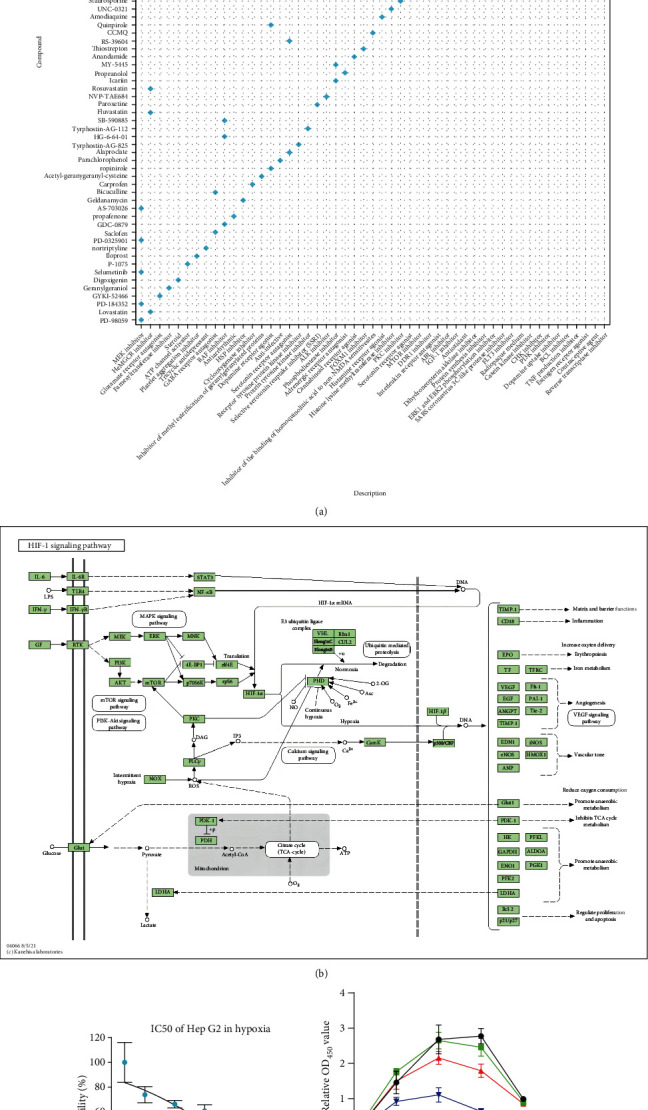
The MEK inhibitor PD-184352 was found and verified as a potential drug to reverse nonresponse to TACE. (a) Compounds predicted by CMap could produce opposite responses to those in the high-risk group in GSE14520. The *x*-axis shows the categories of the compounds, and the *y*-axis shows compound names. (b) Schematic diagram of the HIF-1 signaling pathway. (c) IC_50_ of PD-184352 in Hep G2 was detected in a hypoxic environment. (d) Cell proliferation using lobaplatin at cell-specific IC_50_, PD-184352 at cell-specific IC_50_, or both was measured in a simulated TACE environment *in vitro*.

To find a way to break nonresponse to TACE, we determined DEGs between the high- and low-risk groups. We found that 114 genes were significantly upregulated in the high-risk group (Supplementary file 1, |log_2_FC| > 0.5, *P* < 0.05). We input them into CMap to identify compounds that could potentially reverse nonresponse to TACE. We paid particular attention to drugs that might elicit the opposite response in the human hepatoma cell line Hep G2, because in this study, we used Hep G2 as the TACE nonresponse-like cell line. A total of 69 chemicals with connectivity scores of less than −98 were detected (Supplementary file 2). We found that MEK inhibitors ranked first, indicating that they might reverse nonresponse to TACE (Figure 8(a)). Interestingly, MEK is just upstream of the HIF-1 signaling pathway (Figure 8(b)). Among MEK inhibitors, PD-184352 ranked second and it is easily available; therefore, we identified PD-184352 as a potential reverser of nonresponse to TACE. Subsequently, we detected the IC_50_ of PD-184352 on Hep G2 in a hypoxic environment, which was about 0.43 *μ*M (Figure 8(c)).

Finally, we used lobaplatin and 1% O_2_ to simulate TACE *in vitro* and added PD-184352 to observe its effect on the efficacy of TACE. As shown in Figure 8(d), one-way analysis of variance (ANOVA) showed that cell proliferation of the responder-like cell line Hep G2 was significantly reduced by using either lobaplatin alone (*P* = 0.03) or PD-184352 alone (*P* < 0.0001). When PD-184352 and lobaplatin were combined, the inhibitory effect on cell proliferation was further enhanced (*P* < 0.0001).

## 4. Discussion

With the increasing emphasis on personalized treatment and the lower cost of genome sequencing, the era of precision medicine has arrived. Specifying treatment options and selecting drugs according to genome sequencing results have become a standardized treatment model. In this study, we established a prognostic signature specific to TACE using three genes according to the expression pattern of DEGs between TACE nonresponders and responders, which we verified in cell experiments. More importantly, we selected PD-184352, an inhibitor of MEK, as a drug that could potentially reverse nonresponse to TACE.

The difference between TACE and traditional treatment is that TACE embolizes the blood supply artery when chemotherapeutic drugs are administered. Therefore, the current research has focused on hypoxia- and angiogenesis-related pathways so as to explore differences in TACE response among individuals. Some studies have found that levels of hypoxia-related biomarkers before TACE, such as vascular endothelial growth factor (VEGF) and HIF-1*α*, are negatively correlated with survival and prognosis [6, 20, 21]. HIF-1*α* has been shown to be a key molecule in nonresponse to TACE. In 2018, Liu et al. [22] found that knocking down HIF-1*α* enhanced the efficacy of transarterial embolization. Therefore, the HIF-1 signaling pathway, to which HIF-1*α* belongs, is worthy of in-depth research. In this study, we found that this pathway was significantly enriched in TACE nonresponders compared with TACE responders. Then, using univariate Cox regression analysis, we found that eight genes were related to OS probability in GSE14520. The final prognostic signature was composed of three genes: *EPO*, *HMOX1*, and *SERPINE1*. The EPO protein encoded by the *EPO* gene, mainly expressed in the liver and kidneys, is a hypoxia-reactive protein. A hypoxic environment increases the activity of HIF-1*α*, which binds to cis-acting deoxyribonucleic acid (DNA) hypoxia-response elements (HREs) to activate *EPO* transcription [23]. Much evidence has shown that high expression of *EPO* is associated with poor prognosis in HCC and breast cancer [23, 24], but its role in nonresponse to TACE is still unknown. In view of the important role of *EPO* in hypoxia, we think that it deserves further studies. Heme oxygenase (HO) is the rate-limiting enzyme in heme metabolism [25]. *HMOX1* is a downstream target of the transcription factor HIF-1*α*-limiting enzyme in heme metabolism [26]. The role of *HMOX1* in cancer remains controversial, and further exploration is urgently needed. *SERPINE1*, also known as plasminogen activator inhibitor-1 (*PAI-1*), has long been considered an important factor in poor prognosis of cancer [27, 28]. In 2015, Divella et al. [29] directly proved that high expression of *SERPINE1* is related to poor prognosis in TACE. The functions of these genes, especially their effects on TACE nonresponse, also need further research.

In this study, we obtained the transcriptome sequencing data of liver cancer cell lines in the CCLE database. We treated Hep G2 as a TACE nonresponse-like cell line and Hep 3B as a TACE response-like cell line. Then, we used lobaplatin and 1% O_2_ culture to simulate a TACE environment. We found that Hep G2 cells were more tolerant of the toxic effects of lobaplatin than Hep 3B cells in both hypoxic and normoxic environments. More interestingly, the IC_50_ of Hep G2 was higher in the hypoxic than in the normoxic environment.

We queried CMap build 1.0 based on an L1000 assay using the genes that were significantly upregulated in the GSE14520 high-risk group and screened the MEK inhibitor PD-184352 on the L1000 platform as a drug that could reverse nonresponse to TACE. MEK, also known as MAP2K, regulates HIF-1*α* through the MEK/ERK/HIF-1*α* axis and is an important regulatory factor of the HIF-1 signaling pathway [30, 31]. PD-184352 could simultaneously inhibit the activities of MEK1 and MEK2. Previous studies have demonstrated that PD-184352 shows antitumor activity in thyroid cancer and lung adenocarcinoma [32, 33], but its role in HCC has not been studied. We demonstrated that the combination of lobaplatin and PD-184352 inhibited cell line proliferation more than either treatment alone under hypoxic conditions; therefore, this combination could become a novel intraoperative or postoperative TACE medication strategy. Finally, because we chose only lobaplatin to mimic TACE in our *in vitro* model in this study, we cannot rule out that the use of different chemotherapeutic agents combined with MEK inhibition might have different effects. Our future work will be devoted to explaining the principle underlying clinical application of MEK inhibitors to TACE and providing a theoretical basis for such application.

## 5. Conclusion

We constructed a TACE-specific three-gene signature that could be used to predict HCC patients' responses to and prognosis after TACE treatment. PD-184352 might have potential as a drug to improve TACE efficacy.

## Figures and Tables

**Table 1:** RT-PCR primer sequences.

Gene	Primers
*β*-Actin	F: CACCATTGGCAATGAGCGGTTC
	R: AGGTCTTTGCGGATGTCCACGT
EPO	F: ATGTGGATAAAGCCGTCAGTG
	R: AAAGTGTCAGCAGTGATTGTTC
HMOX1	F: CCTCCCTGTACCACATCTATGT
	R: GCTCTTCTGGGAAGTAGACAG
SERPINE1	F: AACGTGGTTTTCTCACCCTAT
	R: CAATCTTGAATCCCATAGCTGC

**Table 2:** Univariate and multivariate analyses of overall survival in GSE14520.

Factors	Univariate analysis		Multivariate analysis	
	Hazard ratio (95% CI)	*P*	Hazard ratio (95% CI)	*P*
Age (≤50, >50)	1.068 (0.586-1.944)	0.831		
Main tumor size (≤5 cm, >5 cm)	1.878 (1.031-3.421)	**0.040**	1.664 (0.582-4.755)	0.342
Multinodular (yes, no)	1.172 (0.578-2.380)	0.660		
Cirrhosis (yes, no)	7.149 (0.983-51.988)	0.052	10.395 (0.669-161.544)	0.094
TNM staging (I, II, and III)	2.923 (1.539-5.551)	**0.001**	1.212 (0.336-4.369)	0.769
BCLC staging (0 and A, B and C)	2.756 (1.454-5.224)	**0.002**	0.871 (0.297-2.549)	0.801
AFP (>300 ng/mL, ≤300 ng/mL)	0.721 (0.396-1.313)	0.285		
Recurrence months (≤24 months, >24 months)	0.124 (0.057-0.271)	**<0.001**	0.113 (0.046-0.276)	**<0.001**
Risk score	3.732 (2.415-5.769)	**<0.001**	2.109 (1.287-3.455)	**0.003**

## Data Availability

The transcriptome in this research is available in GEO database (https://www.ncbi.nlm.nih.gov/geo/) under accession numbers GSE14520 and GSE104580. The cell line transcriptome in this research is available in CCLE database (https://sites.broadinstitute.org/ccle).

## Supplementary Materials

Supplementary Materials Supplementary file 1: DEGs between the high- and low-risk groups in GSE14520. Supplementary file 2: potential drugs identified by CMap database that might elicit the opposite response in the human hepatoma cell line Hep G2.

## References

[1] Gao Q., Zhu H., Dong L. (2019). Integrated proteogenomic characterization of HBV-related hepatocellular carcinoma. *Cell*.

[2] Kudo M., Ueshima K., Ikeda M. (2020). Randomised, multicentre prospective trial of transarterial chemoembolisation (TACE) plus sorafenib as compared with TACE alone in patients with hepatocellular carcinoma: TACTICS trial. *Gut*.

[3] Liu S., Liu K. C., Lv W. F. (2021). The efficacy and prognostic factors of the combination of TACE and apatinib for the treatment of BCLC stage C hepatocellular carcinoma. *Front Med (Lausanne)*.

[4] Varghese J., Kedarisetty C., Venkataraman J. (2017). Combination of TACE and sorafenib improves outcomes in BCLC stages B/C of hepatocellular carcinoma: a single centre experience. *Annals of Hepatology*.

[5] Peck-Radosavljevic M., Kudo M., Raoul J.-L. (2018). Outcomes of patients (pts) with hepatocellular carcinoma (HCC) treated with transarterial chemoembolization (TACE): Global OPTIMIS final analysis: Global OPTIMIS Final Analysis. *Journal of Clinical Oncology*.

[6] Fako V., Martin S. P., Pomyen Y. (2019). Gene signature predictive of hepatocellular carcinoma patient response to transarterial chemoembolization. *International Journal of Biological Sciences*.

[7] Rankin E. B., Giaccia A. J. (2016). Hypoxic control of metastasis. *Science*.

[8] Lin Z. H., Jiang J. R., Ma X. K. (2021). Prognostic value of serum HIF-1*α* change following transarterial chemoembolization in hepatocellular carcinoma. *Clinical and Experimental Medicine*.

[9] Huang M., Wang L., Chen J. (2016). Regulation of COX-2 expression and epithelial-to-mesenchymal transition by hypoxia-inducible factor-1*α* is associated with poor prognosis in hepatocellular carcinoma patients post TACE surgery. *International Journal of Oncology*.

[10] Piscaglia F., Ogasawara S. (2018). Patient selection for transarterial chemoembolization in hepatocellular carcinoma: importance of benefit/risk assessment. *Liver Cancer*.

[11] Kadalayil L., Benini R., Pallan L. (2013). A simple prognostic scoring system for patients receiving transarterial embolisation for hepatocellular cancer. *Annals of Oncology*.

[12] Xu Y. J., He M. K., Liu S. (2021). Construction of a single nucleotide variant score-related gene-based prognostic model in hepatocellular carcinoma: analysis of multi-independent databases and validation in vitro. *Cancer Cell International*.

[13] Ritchie M. E., Phipson B., Wu D. (2015). Limma powers differential expression analyses for RNA-sequencing and microarray studies. *Nucleic Acids Research*.

[14] Yu G., Wang L.-G., Han Y., He Q.-Y. (2012). clusterProfiler: an R package for comparing biological themes among gene clusters. *Integrative Biology*.

[15] Wang J., Fan W., Ye J. (2015). Fused Lasso screening rules via the monotonicity of subdifferentials. *IEEE Transactions on Pattern Analysis and Machine Intelligence*.

[16] Xiang Z. J., Wang Y., Ramadge P. J. (2017). Screening tests for Lasso problems. *IEEE Transactions on Pattern Analysis and Machine Intelligence*.

[17] Subramanian A., Narayan R., Corsello S. M. (2017). A next generation connectivity map: L1000 platform and the first 1,000,000 profiles. *Cell*.

[18] Kappenberg F., Rahnenführer J. (2020). (2019). Dose-response analysis using R C. Ritz, S. M. Jensen, D. Gerhard, J. C. Streibig Eds, Boca Raton, FL: CRC press, 214 pages. ISBN: 97838431. *Biometrical Journal*.

[19] Ginestet C. (2011). C. ggplot2: Elegant Graphics for Data Analysis. *Journal of the Royal Statistical Society*.

[20] Poon R. T., Lau C., Yu W. C., Fan S. T., Wong J. (2004). High serum levels of vascular endothelial growth factor predict poor response to transarterial chemoembolization in hepatocellular carcinoma: a prospective study. *Oncology Reports*.

[21] Tong C., Liu H., Chen R., Zhu F. (2021). The effect of TACE in combination with thalidomide-mediated adjuvant therapy on the levels of VEGF and bFGF in patients with hepatocellular carcinoma. *American Journal of Translational Research*.

[22] Liu Q., Fan D., Adah D. (2018). CRISPR/Cas9-mediated hypoxia inducible factor-1*α* knockout enhances the antitumor effect of transarterial embolization in hepatocellular carcinoma. *Oncology Reports*.

[23] Yang Z., Sun B., Zhao X. (2015). Erythropoietin and erythropoietin receptor in hepatocellular carcinoma: correlation with vasculogenic mimicry and poor prognosis. *International Journal of Clinical and Experimental Pathology*.

[24] Badowska-Kozakiewicz A. M., Sobol M., Patera J. (2017). Expression of multidrug resistance protein P-glycoprotein in correlation with markers of hypoxia (HIF-1*α*, EPO, EPO-R) in invasive breast cancer with metastasis to lymph nodes. *Archives of Medical Science: AMS*.

[25] Zou C., Zhang H., Li Q. (2011). Heme oxygenase-1: a molecular brake on hepatocellular carcinoma cell migration. *Carcinogenesis*.

[26] Dunn L. L., Kong S. M. Y., Tumanov S. (2021). Hmox1 (heme oxygenase-1) protects against ischemia-mediated injury via stabilization of HIF-1*α* (hypoxia-inducible factor-1*α*). *Arteriosclerosis, Thrombosis, and Vascular Biology*.

[27] Placencio V. R., DeClerck Y. A. (2015). Plasminogen activator inhibitor-1 in cancer: rationale and insight for future therapeutic testing. *Cancer Research*.

[28] Look M. P., van Putten W. L., Duffy M. J. (2002). Pooled analysis of prognostic impact of urokinase-type plasminogen activator and its inhibitor PAI-1 in 8377 breast cancer patients. *Journal of the National Cancer Institute*.

[29] Divella R., Daniele A., Abbate I. (2015). Circulating levels of PAI-1 and SERPINE1 4G/4G polymorphism are predictive of poor prognosis in HCC patients undergoing TACE. *Translational Oncology*.

[30] Gong J., Zhou S., Yang S. (2019). Vanillic acid suppresses HIF-1*α* expression via inhibition of mTOR/p70S6K/4E-BP1 and Raf/MEK/ERK pathways in human colon cancer HCT116 cells. *International Journal of Molecular Sciences*.

[31] Wang G., Li Y., Yang Z., Xu W., Yang Y., Tan X. (2018). ROS mediated EGFR/MEK/ERK/HIF-1*α* loop regulates glucose metabolism in pancreatic cancer. *Biochemical and Biophysical Research Communications*.

[32] Henderson Y. C., Ahn S. H., Clayman G. L. (2009). Inhibition of the growth of papillary thyroid carcinoma cells by CI-1040. *Archives of Otolaryngology--Head & Neck Surgery*.

[33] Kramer B. W., Götz R., Rapp U. R. (2004). Use of mitogenic cascade blockers for treatment of C-Raf induced lung adenoma in vivo: CI-1040 strongly reduces growth and improves lung structure. *BMC Cancer*.

